# Adipose tissue macrophages impair preadipocyte differentiation in humans

**DOI:** 10.1371/journal.pone.0170728

**Published:** 2017-02-02

**Authors:** Li Fen Liu, Colleen M. Craig, Lorna L. Tolentino, Okmi Choi, John Morton, Homero Rivas, Samuel W. Cushman, Edgar G. Engleman, Tracey McLaughlin

**Affiliations:** 1 Division of Endocrinology, Department of Medicine, Stanford University, Palo Alto, California, United States of America; 2 Stanford Blood Center, Palo Alto, California, United States of America; 3 Department of Surgery, School of Medicine, Stanford University, Palo Alto, California, United States of America; 4 National Institute of Diabetes and Digestive and Kidney Diseases, National Institutes of Health, Bethesda, Maryland, United States of America; 5 Department of Pathology, School of Medicine Stanford University, Palo Alto, California, United States of America; Universita degli Studi di Bari Aldo Moro, ITALY

## Abstract

**Aim:**

The physiologic mechanisms underlying the relationship between obesity and insulin resistance are not fully understood. Impaired adipocyte differentiation and localized inflammation characterize adipose tissue from obese, insulin-resistant humans. The directionality of this relationship is not known, however. The aim of the current study was to investigate whether adipose tissue inflammation is causally-related to impaired adipocyte differentiation.

**Methods:**

Abdominal subcutaneous(SAT) and visceral(VAT) adipose tissue was obtained from 20 human participants undergoing bariatric surgery. Preadipocytes were isolated, and cultured in the presence or absence of CD14+ macrophages obtained from the same adipose tissue sample. Adipocyte differentiation was quantified after 14 days via immunofluorescence, Oil-Red O, and adipogenic gene expression. Cytokine secretion by mature adipocytes cultured with or without CD14+macrophages was quantified.

**Results:**

Adipocyte differentiation was significantly lower in VAT than SAT by all measures (p<0.001). With macrophage removal, SAT preadipocyte differentiation increased significantly as measured by immunofluorescence and gene expression, whereas VAT preadipocyte differentiation was unchanged. Adipocyte-secreted proinflammatory cytokines were higher and adiponectin lower in media from VAT vs SAT: macrophage removal reduced inflammatory cytokine and increased adiponectin secretion from both SAT and VAT adipocytes. Differentiation of preadipocytes from SAT but not VAT correlated inversely with systemic insulin resistance.

**Conclusions:**

The current results reveal that proinflammatory immune cells in human SAT are causally-related to impaired preadipocyte differentiation, which in turn is associated with systemic insulin resistance. In VAT, preadipocyte differentiation is poor even in the absence of tissue macrophages, pointing to inherent differences in fat storage potential between the two depots.

## Introduction

While the link between obesity and insulin resistance in humans has been clearly established, the causal basis for this association is unclear. Not all obese are metabolically unhealthy and even normal-weight individuals can be insulin resistant: thus there are likely qualitative differences in adipose tissue that contribute to metabolic risk independent of adiposity *per se*. We and others have hypothesized that in the setting of excess body weight, impaired differentiation of new adipocytes leads to hypertrophy of existing adipocytes and spillover of fat into non-adipose tissues, thereby contributing to insulin resistance via lipotoxicity[1–4]. Furthermore, hypertrophic adipocytes in subcutaneous adipose tissue (SAT) appear to attract inflammatory cells, as histologic sections reveal rings of macrophages surrounding single enlarged necrotic adipocytes [5]. Increased subcutaneous adipocyte diameter has also been associated with insulin resistance and type 2 diabetes [6,7]. Finally, increasing evidence suggests that preferential deposition of fat in visceral adipose tissue (VAT) is associated with metabolic abnormalities such as insulin resistance, dyslipidemia, fatty liver, and type 2 diabetes [8–10]. While VAT comprises only approximately 10% of total adipose tissue mass [11], it is characterized by increased inflammation relative to SAT [12], which might explain its link to systemic insulin resistance [13].

Whether adipocyte dysfunction and hypertrophic adipose cells initiate an inflammatory response or inflammation in adipose tissue is a primary event that impairs adipocyte differentiation is unknown. The latter possibility is supported by in vitro studies showing that when 3T3-L1adipocytes are cultured with RAW 264 macrophages or cytokines, differentiation and triglyceride uptake is decreased and adipocytes adopt a proinflammatory phenotype [3,14] Further, isolated human preadipocytes do not differentiate normally in the presence of cytokines or macrophage-conditioned media prepared from cultured blood monocytes or adipose tissue macrophages [14], and inflammatory cytokines such as tumor-necrosis factor-alpha (TNFα) have been shown to directly impair insulin action in animal models [15]. If this were the case, targeted treatments to decrease inflammation in adipose tissue might improve metabolic manifestations related to adipose tissue dysfunction such as increased lipolysis, circulating free fatty acids, ectopic fat deposition, and insulin resistance. We thus designed a study to test the hypothesis that inflammation in adipose tissue impairs differentiation of human preadipocytes by co-culturing preadipocytes isolated from SAT and VAT of healthy obese human subjects, with and without macrophages isolated from the same tissue samples.

## Materials and methods

### Subjects

Healthy obese participants were recruited sequentially from the Stanford University Bariatric Surgery Clinic. Eligibility requirements included age >25 yrs, BMI ≥30 kg/m^2^, absence of diabetes as defined by fasting plasma glucose < 126 mg/dL and no use of antidiabetic medications. Further, subjects had to be free of major organ disease, chronic inflammatory conditions, pregnancy/lactation, use of weight loss medications, and change in weight of more than 2 kg during the month before surgery. The protocol was approved by the Stanford Human Subjects Committee, and all subjects gave written, informed consent. Due to limited cell number, subjects were divided into two cohorts who underwent different adipocyte analyses: cohort 1 (n = 9) for comparison of differentiation and inflammation by fat depot, and cohort 2 (n = 10) for evaluation of differentiation with and without removal of proinflammatory CD14+ macrophages Table 1.

**Table 1:** Metabolic characteristics of the study subjects.

Characteristics	Cohort 1	Cohort 2
	N = 9, Mean ± SD	N = 10, Mean ± SD
**Age (yrs)**	49.3 ± 8.7	45.8 ± 11.6
**Sex (F/M)**	7/2	9/1
**Race (C/H/B/A)**	(5/2/2/0)	(5/3/2/0)
**BMI (kg/m2)**	45.8 ± 4.7	44.3 ± 6.8
**Fasting glucose (mmol/L)**	5.55± 1.08	5.26 ± 0.72
**Fasting insulin (uIU/mL)**	16.7 ± 6.7	20.8 ± 12
**HbA1C (%)**	5.87 ± 0.17	5.87 ± 0.58

B: Black; A: Asian; BMI: body mass index; hsCRP: high sensitivity C-reactive protein

C: Caucasian; H: Hispanic;

### Differentiation of human preadipocytes

#### Tissue harvesting

On the day of surgery, approximately 2 to 3g each of subcutaneous abdominal and omental visceral adipose tissue was harvested intraoperatively and immediately processed in our research laboratory: 500 mg was flash frozen for measurement of gene expression and the remainder underwent collagenase digestion for separation of adipocytes and stromal vascular cells. The study was approved by the Stanford University Humans Subjects Committee, and all subjects gave written, informed consent.

#### Adipose tissue digestion and stromal vascular cell separation

Subcutaneous and omental adipose tissue were digested by collagenase I (0.1M HEPES, 0.12M NaCl, 0.05M KCL, 0.005M glucose, 1.5% wt/vol BSA, 1mM CaCl2, 2H2O, pH7.4) at 37°C for 60 min, according to the original protocol of Rodbell as previously described [16]. The digested tissue was then filtered through a 250-um nylon mesh and centrifuged for 5 min at 500 g at room temperature (RT). Mature fat cells were removed by flotation and utilized for adipocytokine assay per below. The stromal vascular cells (SVC) were separated by further centrifugation at 800 g at RT. The resulting cell pellet was incubated in erythrocyte lysis buffer (ACK erythrocyte lysis buffer, invitrogen) for 10 min at 37°C. PBS with 2% BSA was added to the cell suspension following erythrocyte lysis procedure. The purified stromal vascular cells then centrifuged for another 5 min at 850 g at RT. Cell pellets were filtered through a 75-um cell strainer after washing with 2% PBS buffer twice.

#### Differentiation of preadipocytes isolated from SAT and VAT

SVCs were isolated from SAT and VAT of 9 participants during bariatric surgery (**Cohort 1**) Table 1. Differentiation of human preadipocytes was induced with a differentiation media (Zenbio,Inc) consisting insulin, dexamethasone and isobutylmethylxanthine (IBMX) and pioglitazone supplemented with 10% FBS. After 3 days, the media was changed to adipocyte maintenance media (Zenbio, Inc) containing only insulin, dexamethasone in DMEM/F12 supplemented with 10% FBS (Fetal bovine serum). The cells were then left to differentiate for another 11–12 days with medium changed every other day.

#### Depletion of CD14+ cells

SVCs were collected from SAT and VAT of 10 participants during bariatric surgery (**Cohort 2**) as described above Table 1. After the separation of SVCs, proinflammatory macrophages were removed via immunomagnetic depletion of human CD14+ cells using magnetic beads (Human CD14 Selection kit Cattalog#18058, Stem Cell Technologies Inc, Canada). Efficacy of CD14 removal was evaluated by flow cytometery analysis using CD14 (APC-H7) and CD45 (V450) antibodies, demonstrating removal of 97 percent of CD14+ cells (representative plots of gated CD45+ and CD14- population shown in S1a Fig. To characterize the CD14+ cell population in SVC isolated from human adipose tissue, FACS analysis was performed on the freshly isolated cells from SVC using CD206-APC and CD163-PE. All CD14+ cells expressed both CD206 and CD163, macrophage cell surface markers (S1a and S1c Fig).

#### Co-culture of CD14+ cell with preadipocytes

To control for starting number of preadipocytes for each patient, after CD14 depletion, cells were counted in the CD14- SVC pellet and plated at a concentration of 100,000 cells / **4 cm**^**2**^, and then co-cultured with or without the CD14+ cells (1:100) by adding separated CD14 + cells directly into SVC culture in a 12 well culture plate at day 0. Cells cultured with or without CD14 were then left on the plate with changing media every other days until reached near 100% confluence at 7. SVC with or without CD14+ co-culture were then induced to differentiate at day 7 with a differentiation media (Zenbio,Inc) consisting insulin, dexamethasone and isobutylmethylxanthine (IBMX) and pioglitazone supplemented with 10% FBS. After 3 days, the media was changed to adipocyte maintenance media (Zenbio, Inc) containing only insulin, dexamethasone in DMEM/F12 supplemented with 10% FBS (Fetal bovine serum). The cells were then left to differentiate for another 11–12 days with medium changed every other day. The CD14 cells for co-culture were freshly isolated from the same adipose tissue sample as the preadipocytes, and not from peripheral blood or pooled samples.

### Immunofluorescence analysis of adipocyte differentiation

Preadipocytes from day 14 of differentiation were stained with Hoechst-33258 (2 ug/mL, Molecular probes, Invitrogen) for cell nuclei (blue), and Bodipy (1:500 of 1 mg/dL stock) for lipid droplets (green). Images of fluorescently labeled cells were examined immediately on an inverted fluorescent microscope (Leica CTR 6500). Merged images of cells co-stained with Hoechest (nuclei) and Bodipy (lipid) were counted by Image J. At least 800–1000 cells per donor, as determined by numbers of nuclei (blue), were counted on three to four high power fields using image analysis software (Image J). Percentage (%) of differentiated adipocytes was calculated as: (Hochest+ Bodipy+)] /(Hochest+)] x100. Calculations as described were measured at day 14 of differentiation.

### Oil Red O staining of lipid droplets

To quantify lipid droplet formation in adipocytes, on day 14 of differentiation adipocytes were fixed in formalin (10%) for 20 min, washed with dH2O, and then stained with Oil Red O for 20 min. After staining, cells were washed with dH2O, followed by an additional three washes (5 min each) with 60% isopropanol. Oil Red O stain was extracted with 100% isopropanol for 5 min. Quantification of lipids was performed by optical density measurement at 492 nm.

### Quantification of relative gene expression in human adipocytes with and without CD14+macrophages

On day 14 of culture, total RNA was extracted from the differentiated human adipocytes derived from SAT and VAT, both with and without CD14+ macrophage removal, using TriZol (Invitrogen, Carlsbad, CA, USA) and the Adipose Tissue RNAeasy kit (Qiagen, Valencia, CA, USA) according the manufacturer’s instructions. cDNA was synthesized from 1 ug of total RNA using High Capacity RNA-to-cDNA kit (Applied Biosystems. Carlsbad, CA, USA). Taqman primers/probes set for gene expression of PPARγ, CEBPβ, adiponectin and IL-6 were purchased from Applied Biosystems (Carlsbad, CA, USA). GAPDH was utilized as a house keeping gene. Validation of endogenous reference genes for qRT-PCR analysis of human visceral adipose has been previously shown by Mehta et al [17]. Amplification was carried out in triplicate and multiplex on an ABI Prism 7900 Sequence Detection System (Applied Biosystems (Carlsbad, CA, USA). A threshold cycle (Ct value) was obtained from each amplification curve, and a Δ Ct value was first calculated by subtracting the Ct value for 18S ribosomal RNA from the Ct value for each sample. A ΔΔCt value was calculated by subtracting the Δ Ct value of a SAT adipocyte sample (control). Fold changes compared with the control were then determined by using the comparative threshold method (2 − ΔΔCt) [18].

### Cell proliferation assay

In order to be sure that differences observed in adipocyte differentiation were not due to differences in cell proliferation, proliferation in the SVC fragment was analyzed using BrdU (bromodeoxyuridine) incorporation assay (S2 Fig) at day 2 and day 7. Stromal vascular cells derived from subcutaneous and omental adipose tissues were seeded onto a 96-well culture plate, cells were plated at 2 ×10^5^ cell/mL in 100uL/well of appropriate cell culture media. Some of the wells on the plate were set aside for the following controls including 1) blank: tissue culture supernatant only (no cells) 2) background: cells are present in the wells but without added BrdU reagent. BrdU, a synthetic thymidine analogue, can be incorporated into newly synthesized DNA providing a test of DNA replication, as an indirect measure of cell division. The assay was performed as described in the product manual from Millipore. BrdU incorporation was detected by addition of peroxidase substrate. Spectrophotometric detection was performed at a wavelength of 450 nm.

### Determination of adipocytokine concentrations by Luminex

Mature human adipocytes derived from subcutaneous and omental adipose tissue were separated by collagenase digestion and flotation as described above: mature adipocytes were then co-cultured with or without CD14+ macrophages isolated from the same adipose tissue sample (and therefore same depot and same individual) (1:100). After culturing for 14 hours, supernatants obtained from co-culture media were collected and analyzed for adipocytokines including adiponectin, interleukin-6 (IL6), and monocyte chemoattractant protein-1 (MCP-1), measured using a multiplex human adipokine assay (Human Adipocyte Panel, Millipore, Bedford, MA, USA), and detected by Luminex xMAP (Luminex 200, Millipore, Bedford, MA, USA).

### Statistical analysis

All variables were normally distributed. Dependent variables of interest (percent differentiation, adipocytokines and gene expression) in SAT versus VAT were compared by Student’s paired t-test. Comparison of changes within groups resulting from CD14 exposure also utilized Student’s paired t-test. Correlational analyses utilized multiple linear regression analysis with adjustment for BMI. Data were expressed as mean ± standard deviation (SD), and P<0.05 was considered statistically significant. Due to small sample size, p<0.10 was noted in the text.

## Results

### Subjects

Nineteen participants, consisting of 16 females and three males, were recruited (Table 1). Subjects were of primarily Caucasian ethnicity, with average age of 48 years and BMI of 44.3±6.8 kg/m2. Cohort1 and Cohort 2 were similar with regard to their demographic and clinical characteristics.

### Depot-specific preadipocyte differentiation

As shown in Table 2, Cohort 1 demonstrated markedly decreased preadipocyte differentiation in VAT as compared with SAT, as reflected in the % differentiated adipocytes measured by immunofluorescence (p<0.001), Oil Red O lipid extraction (p < 0.001), and gene expression (adiponectin p = 0.002, PPARG p = 0.07). In Cohort 2, (Table 3) differentiation by immunofluorescence and gene expression were measured at day 14 in two subgroups: adipocytes differentiated in culture with and without macrophages from the same tissue sample from which the preadipocytes were isolated. In the presence of adipose tissue macrophages, SAT preadipocyte differentiation was greater than VAT, as measured by immunofluorescence (p = 0.0007) and gene expression (adiponectin p = 0.03, PPARG p = 0.026); in the absence of adipose tissue macrophages, SAT preadipocyte differentiation was still markedly greater than VAT preadipocyte differentiation (immunofluorescence p<0.00001, adiponectin p = 0.004, PPARG p = 0.01).

**Table 2:** Adipocyte differentiation capacity in subcutaneous (SAT) versus visceral (VAT) adipose tissue: Immunofluorescence, Oil Red O, and gene expression in preadipocytes after 14 days in culture (mean ± SD) (Cohort 1, n = 9).

Variable	SAT	VAT	p-value
% Differentiation by immunofluorescence^#^	60.0 ± 14.7*	13.9 ± 7.3	0.001
Oil Red extraction (OD)	78 ± 5.6*	6.7 ± 2.1	0.001
Relative Gene Expression in Differentiated Human Adipocytes			
Adiponectin	1.012 ± 0.34	0.04 ± 0.07	0.002
PPAR-γ	2.29 ± 0.71	0.94 ± 0.62	0.07
CEBP-β	1.79 ± 25	1.64 ± 34	0.82
IL-6	0.99 ± 0.39	0.85 ± 0.22	0.32

^#^: [(Hoescht+bodipy+) / Hoescht+]×100,

*p< 0.05 SAT vs VAT.

**Table 3:** Adipocyte differentiation in subcutaneous (SAT) and visceral (VAT) adipose tissue after removal of CD14+ macrophages: Immunofluorescence and gene expression in preadipocytes after 14 days in culture (Cohort 2, n = 10).

Adipose depot	SAT		VAT		p-value			
Variable	CD14-	CD14+	CD14-	CD 14+	p-value*	p–value**	p-value #	p-value##
Differentiation ^$^	57.7 ± 12.4*	39.7 ± 12.3**	11.0 ± 4.4	10.8 ± 5.8	0.018	0.0007	0.00001	0.588
**Relative Gene Expression in differentiated human adipocyte**								
Adiponectin	1.08 ± 0.28*	0.47 ± 0.37**	0.12 ± 0.23#	0.10 ± 0.20	0.001	0.03	0.0004	0.88
PPAR-γ	1.26 ± 0.37	0.95 ± 0.48**	0.46 ± 0.31#	0.42 ± 0.25	0.153	0.026	0.01	0.809
CEBPβ	1.12 ± 0.13*	0.73 ± 0.25	0.95 ± 0.43	0.72 ± 0.42	0.02	0.958	0.35	0.367
GLUT4	0.96 ± 0.18*	0.52 ± 0.33	0.48 ± 0.50	0.25 ± 0.30	0.004	0.129	0.09	0.36
IL6	1.10 ± 0.16*	1.76 ± 0.58	1.049 ± 0.32	1.82 ± 1.02	0.02	0.88	0.76	0.129

^$^ % Differentiation by immunofluorescence[(Hoescht+bodipy+) /Hoescht+] × 100,

*p< 0.05 SAT CD14- vs SAT CD14+,

**p<0.05 SAT CD14+ vs. VAT CD14+

^#^ *P* < 0.05 SAT CD14- vs. VAT CD14-.

^##^ p<0.05 VAT CD14+ vs. VAT CD14+

### Effect of macrophage removal on human adipocyte differentiation

**Fig 1:**
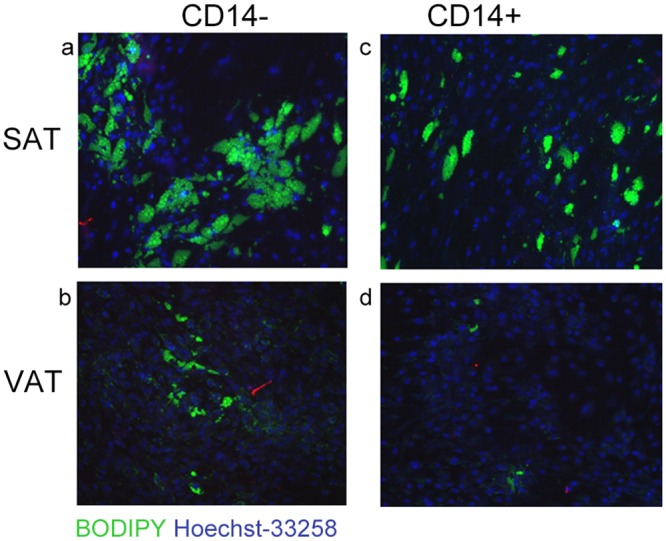
Immunofluorescence of differentiated human adipocytes. Representative immunestaining of lipid droplets (BODIPY, green fluorescence) counterstained with Hoechst-33258 (blue fluorencence) of differentiated preadipocytes from human SAT (a, c) and VAT (b, d) with and without CD14+ cells; magnification X20

Comparison of preadipocyte differentiation in the presence or absence of macrophages from the same tissue sample was conducted for both SAT and VAT. Fig 1 depicts differentiation assessed by immunofluorescence, in which differentiated cells containing lipid droplets (green) are considered relative to total number of viable cells with stained nuclei (blue) on day 14 of differentiation. As shown in Table 3, the number of differentiated cells from VAT was not significantly changed by co-culturing with or without macrophages. In SAT, however, adding back the macrophages to the differentiating preadipocyte culture led to a 31% decrease in adipocyte differentiation (p = 0.018), as measured by immunofluorescence. Similarly, expression of differentiation genes (adiponectin, CEBPβ, GLUT4) by adipocytes after 14 days of differentiation was significantly decreased in the co-culture with macrophages in SAT. Furthermore, IL6 expression increased significantly in adipocytes co-cultured with macrophages in SAT.

### Effect of CD14+ macrophage on preadipocyte proliferation

In order to rule out the possibility that differences in proliferation contributed to observed differences in preadipocyte differentiation, BrdU proliferation assay was performed on SVC derived from a subset (n = 5) of the total cohort at two different days prior to differentiation. Cell proliferation as indicated by BrdU incorporation was significantly greater in SAT as compared with VAT (p < 0.05) at day 2 (S2a Fig). Cell proliferation was not altered by macrophage exposure in either depot at day 7 prior to induction of differentiation (S2b Fig).

### Macrophage effects on adipocytokine secretion by mature adipocytes

**Fig 2:**
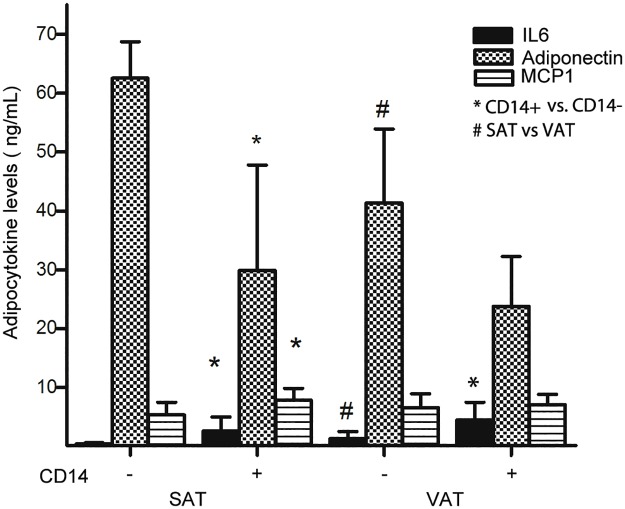
IL6, adiponectin and MCP-1 levels in the media of human fat cells from SAT or VAT cultured with or without CD14+ macrophages. Data expressed as mean ± SE. * *P* < 0.05 CD14- vs CD14+ (SAT and VAT), # *p* <0.05 SAT vs VAT.

To explore the possible proinflammatory and anti-differentiation effects of adipose tissue macrophages on human adipocytes, mature adipocytes isolated from SAT and VAT were cultured in the absence (CD14-) or presence (CD14+) of adipose-derived macrophages isolated from the same sample of adipose tissue from which adipocytes were harvested in each subject. In the media of adipocytes from both SAT and VAT, co-culture with adipose tissue macrophages caused a significant decrease in adiponectin concentration and a significant increase in IL6 concentration; in media from SAT only, MCP-1 concentrations increased significantly (Fig 2).

### Relationship between preadipocyte differentiation and insulin resistance

**Fig 3:**
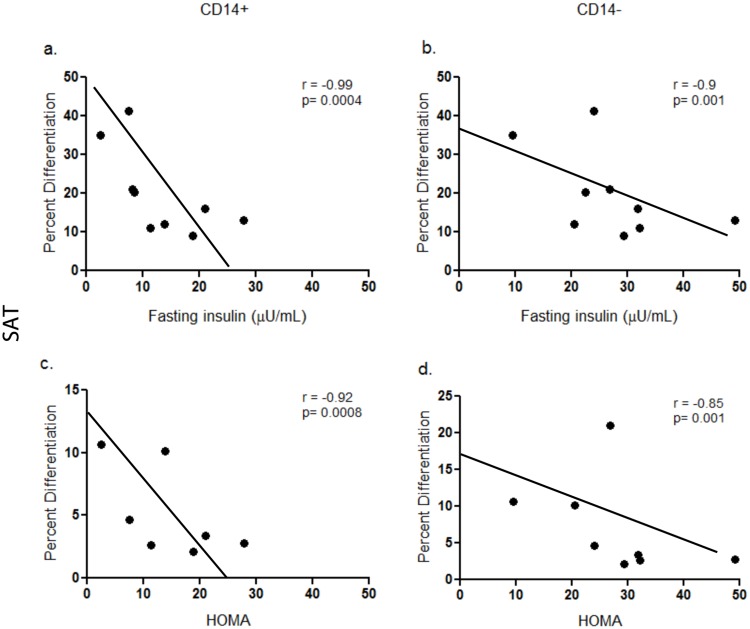
Correlations between human adipocye differentiation and insulin sensitivity. Differentiation of preadipocytes from SAT but not VAT correlated inversely with systemic insulin resistance as measured with HOMA and fasting insulin in the presence or absence of CD14 cells (r = 0.92, p = 0.0008 CD14+, r = 0.85, p = 0.001 CD14-). (a) Correlation between fasting insulin and adipocyte differentiation of SAT CD14+ (b) Correlation between fasting insulin and adipocyte differentiation of SAT CD14- (c) Correlation between HOMA and adipocyte differentiation of SAT CD14+ (d) Correlation between HOMA and adipocyte differentiation of SAT CD14-.

Differentiation of preadipocytes isolated from SAT but not VAT correlated inversely with insulin resistance as measured by HOMA or fasting insulin (Fig 3). This relationship was independent of BMI and was present irrespective of the presence or absence of CD14+ macrophages (CD14+ r = -0.92, p = 0.0008; CD14- r = -0.85, p = 0.001 with HOMA; CD14+ r = -0.99, p = 0.0004; CD14- r = -0.9; p = 0.001), indicating that systemic insulin resistance is associated with poor preadipocyte differentiation independent of CD14+ cells. BMI and fasting glucose were not associated with preadipocyte differentiation.

### Charactrization of macrophage phenotype in SAT and VAT

The composition of M2 vs. M1 in SAT and VAT was further characterized by flow cytometry and gene expression in six subjects. Flow cytometric analysis of SAT vs VAT macrophage markers is shown in S3A Fig. There were no significant differences (paired t-test) between depots in M1 or M2 phenotype, although M2 tended to be higher in VAT than SAT (p = 0.07), and monocytes (CD11c+CD206-) showed a similar trend (p = 0.07). As shown in S3B Fig, gene expression results show a trend of increase in IL6 (P = 0.07) in VAT vs SAT whereas all other M1 and M2 genes tended to be increased in SAT.

## Discussion

The results of this study extend a small but growing body of literature suggesting that inflammation in adipose tissue plays a causal role in adipocyte dysfunction and insulin resistance. While multiple studies in rodents have shown that diet-induced obesity leads to adipocyte hypertrophy [5,15], systemic and localized inflammation [19], and the development of insulin resistance, the directionality of these relationships is unclear. Hypertrophic (versus hyperplastic) obesity, for example, which is presumed to reflect impaired differentiation of new adipocytes to “offload” triglyceride storage demands, correlates with macrophage density in human adipose tissue, and macrophages tend to cluster geographically around single large necrotic adipocytes [5]. Thus, adipocyte hypertrophy may initiate inflammation in adipose tissue. Alternatively, 3T3-L1 adipocytes, when cultured with inflammatory cytokines, exhibit defective terminal differentiation and markedly reduced lipid uptake [2], implying that inflammation may impair adipocyte differentiation and thus, lead to hypertrophy of non-inflamed adipocytes. Inflammatory cytokines also have been shown to directly antagonize insulin action in human adipocytes [14, 20–22], thereby inhibiting fat storage and promoting lipotoxicity.

The current study addresses a major gap in translating ex-vivo studies into humans. Here we provide compelling evidence in support of the hypothesis that inflammation in SAT is causaly related to adipocyte dysfunction and insulin resistance in humans. Specifically, we demonstrated that removal of proinflammatory SAT macrophages significantly enhanced differentiation of preadipocytes from the same tissue sample, increased expression of differentiation genes, and altered secretory profile of isolated adipocytes such that adiponectin was increased and MCP-1 and IL-6 were decreased. These changes were not present in VAT, which maintained a poor differentiation and proinflammatory state despite CD14 macrophage removal. Furthermore, we showed that insulin resistance is highly correlated with differentiation of preadipocytes in SAT, but not in VAT, highlighting an indirect link between adipose tissue inflammation and systemic insulin resistance, via maladaptive effects on adipose cells.

Our results extend those of Isaakson [3], who cultured human subcutaneous preadipocytes with commercially-available inflammatory cytokines, showing marked reduction in differentiation assessed by Oil Red O accumulation and adipogenic gene expression. Current findings also extend those of Lacasa [23], who cultured subcutaneous human preadipocytes in pooled conditioned media from blood monocyte-derived and adipose tissue macrophages, showing decreased lipid accumulation and expression of PPARγ, C/EBPα, and genes reflecting terminal differentiation, and Coutier [24], who demonstrated decreased differentiation of cryopreserved human preadipocytes when co-cultured with peripheal blood mononuclear cells. Our study results more closely reflect in vivo physiology than prior studies because co-cultures were performed with freshly harvested human preadipocytes and CD14+cells from the same adipose tissue sample, thus suggesting that there is a paracrine effect of adipose tissue macrophages on human SAT preadipocytes. Our findings differ somewhat form those of Chazenbalk et al, who showed that adipose tissue macrophages increase preadipocyte formation, and proposed that the macrophages themselved differentiated into preadipocytes [25]. With floating mature fat cells floating in coculture system by Chaenbalk et al might contribute the difference in macrophages differentiation [25]. The current study is also the first to quantify the effects of adipose tissue macrophages on human preadipocytes from VAT. Interestingly, unlike SAT, differentiation of preadipocytes from VAT was unaltered by removal of proinflammatory macrophages. Whether this is due to paracrine effects of other cells in the VAT stromal-vascular fraction, greater proportion of stem cells, high baseline levels of inflammation that were not reversed simply by removal of CD14+ cells, or to inherent differences in the preadipocytes themsleves is not clear, and worthy of further investigation.

The second major finding of this study is the marked between-depot difference in preadipocyte differentiation. This has been shown previously to some degree. Isolated rodent [26] and human [27–28] preadipocytes from VAT as compared to SAT in several small studies showed reduced differentiation potential as well as significant differences in gene expression profiles, implying different biological functions of these two fat depots. Our study extends these findings by showing in human cohort not only that preadipocytes from VAT demonstrate markedly impaired differentiation in culture, but also that VAT differentiation is impaired even when inflammatory CD14 cells are removed. This finding, in combination with adipocyte stem cell (ASC) studies showing that ASC from SAT as compared to VAT demonstrate greater differentiation potential, adiponectin secretion, and reduced susceptibility to lipolysis [29–32], point to intrinsic differences in adipocytes from different depots. **Further, between-depot differences in the proportion of stem cells (which have to divide prior to differentiating) to committed preadipocytes (already in the differentiation pathway) might have contributed to observed differences in differentiation between SAT and VAT (26, 29). Thus, poor differentiation of preadipocytes from VAT as compared to SAT** may not merely reflect the relatively greater degree of inflammation in the visceral tissue depot.

Another important finding from our study is that in the presence of inflammatory cytokines, preadipocytes can take on a proinflammatory phenotype. We have previously shown that expression of inflammatory genes in adipose tissue is highly correlated with the proportion of small adipose cells [1]. It was unclear from this study whether these genes were expressed by immune cells in the adipose tissue or the adipose cells themselves. In the current study, we demonstrated both increased MCP1 and IL6 secretion and decreased adiponectin (antiinflammatory) secretion after co-culture of mature SAT adipocytes with CD14+ macrophages from the same tissue sample. In VAT, the addition of CD14+ cells in culture increased secretion of IL6 and decreased secretion of adiponectin to a lesser degree, and MCP1 did not change significantly. These findings were mirrored by increased RNA expression of IL6 in differentiated adipocytes after co-culture with adipose tissue macrophages in both SAT and VAT preadipocytes, albeit expression change in VAT did not reach statistical significance due to greater variability. The limitation of our conclusion with macrophages present in the culture at the end of cell differentiation might be a potential confounding factor in alteration of adipocyte differentiation. These findings extend those of Gustafson [2] who demonstrated increased inflammatory cytokine secretion from 3T3L1 adipocytes when cultured in the presence of commercial cytokines (TNF-a and IL6), and also of Isaakson, who demonstrated the same in human preadipocytes cultured for 10 days with commercially available TNF-a [3]. Another group showed that when 3T3-L1 adipocytes are co-cultured with monocytes, secretion of TNF-α (from monocytes) stimulates elaboration of IL-6, IL-8, MCP-1, and TNF- α from the adipocytes, a process that is prevented in the presence of TNF-α blocking antibody [33]. Our results bear greater physiologal relevance, however, given that freshly obtained human preadipocytes were cultured with adipose tissue macrophages derived from the same tissue sample, and thus concentrations of cytokines were more likely to represent physiologic exposure levels in human adipose tissue. These data thus suggest that not only do inflammatory macrophages in adipose tissue impair differentiation of preadipocytes, but that they may cause them to adopt a proinflammatory phenotype.

Finally, we have previously hypothesized that impaired adipocyte differentiation may be causally related to insulin resistance [7]. Prior studies in humans to this date have shown associations between differentiation gene expression in adipose tissue and adipocyte cell size and insulin resistance [1,4,6]. The current results demonstrate a robust indirect relationship between preadipocyte differentiation and systemic insulin resistance in SAT. To this date there are no published studies showing functional impairment of preadipocytes differentiation in association with systemic insulin resistance. Interestingly, this was present only in SAT. As described above, it is likely that the function of adipocytes from SAT and VAT differ considerably. Given that SAT represents 90% of total fat mass, it has potential to greatly affect systemic insulin resistance via secretion of cytokines or release of free fatty acids that circulate to muscle where 85% of IMGU, insulin-mediated glucose uptake occurs. Alternatively, VAT may contribute to insulin resistance but not likely via impaired differentiation. Mass of VAT is correlated with insulin resistance in the literature. What is not clear is how the function or qualitative aspects of this depot may contribute beyond mass per se to cardiometabolic risk. Indeed, it is possible that inflammation in VAT is a mediator of insulin resistance, whereas in SAT, which comprises 80–90% of total body fat, differentiation and triglyceride storage are more important determinates of insulin resistance. Our working hypothesis, not addressed per se in this paper, is that SAT’s role is in triglyceride storage and if fat storage is compromised, fatty acids circulate to non-adipose tissues and contribute to insulin resistance. VAT may ever serve as an overflow depot, expanding in mass when fat storage capacity is compromised in the SAT. adipocytes are inherently different in VAT as shown by microarray studies [26], and differentiation in this depo may have a less important role than inflammation/cytokine secretion on systemic insulin resistance.

It is more likely that secretion of proinflammatory cytokines and FFA released from VAT alter insulin sensitivity via direct effects on hepatic secretion of proinflammatory proteins and hepatic lipid and glucose metabolism. We believe that overweight as well as morbidly obese individuals would share similar patterns. We have not studied lean individuals and it is not known how cells would respond in such individuals. The limitation of our conclusions may not extend to lean or healthy overweight individuals.

In conclusion, the results of the current study reveal that proinflammatory macrophages in human SAT impair preadipocyte differentiation and promote a proinflammatory phenotype. VAT preadipocytes differentiate poorly, even in the absence of tissue macrophages, pointing to inherent differences in fat storage potential between the two depots. Impaired adipocyte differentiation in SAT is associated with systemic insulin resistance and thus, targeting macrophage infiltration and/or activity in adipose tissue may represent a new target for treatments aimed at reversing obesity-associated insulin resistance.

## Supporting information

S1 Fig Characterization of the isolated CD14+ cells. (a) Representative FACS analysis perform on SVCs in the presence or absence of CD14 positive cells from human adipose tissue using CD14(APC-H7) and CD45 (V450) antibodies. (b-c) Characterization of the isolated CD14+ cells. To characterize the CD14+ cells population, FACS analysis was performed on the freshly isolated CD14 positive cells from SVC. (b, c) All cells express both CD206 and CD163macrophage specific markers.
(TIF)

S2 Fig Cell proliferation rate of preadipocytes from SAT and VAT cocultured with and without CD14 positive cells. (A) Rate of proliferation of preadipocytes after 2 days of coculture with Cd14+ cells. Rate of proliferation was determined by BrdU incorpartion *N* = 3, error bars are means ± s.d., *P < 0.05 compared to CD14- cells. (B) Rate of proliferation of preadipocytes during coculture with CD14 + cells at day 7 at a 1:100, CD14+ cells: preadipocyte ratio. *N* = 3, error bars are means ± s.d.
(TIF)

S3 Fig Characterization of M2/M1 macrophage phenotype in SAT and VAT. (A) Characterization of M2 and M1 phenotype by flow cytometry in SAT and VAT (N = 6, data are presented as mean ± SD). (B) Relative fold change in gene expression of M2 (ARG1, IL10) and M1 (CD11c, IL6 and CCL2) markers in SAT and VAT (N = 7, data are presented as mean ± SD)
(TIF)

## Abbreviations

ATM: Adipose tissue macrophages
BMI: Body Mass Index
BrdU: Bbromodeoxyuridine
HOMA: Homeostatic model assessment
IBMX: 3-isobutyl-1-methylxanthine
IS: Insulin sensitive
IR: Insulin resistant
IL6: Interleukin 6
MCP-1: Monocyte chemoattractant protein-1
qRT-PCR: Quantitative real-time PCR
SSPG: Steady-state plasma glucose
SAT: Subcutaneous adipose tissue
VAT: Visceral adipose tissue
SVC: Stromal vascular cell
TNFα: Tumor-necrosis factor-alpha

## References

[1] McLaughlin T, Deng A, Yee G, Reaven G, Tsao PS, Cushman SW, et al Inflammation in subcutaneous adipose tissue: relationship to adipose cell size. Diabetologia. 2010; 53:369–77. 10.1007/s00125-009-1496-3 19816674 PMC6290757

[2] Gustafson B, Gogg S, Hedjazifar S, Jenndahl L, Hammarstedt A, Smith U. Inflammation and impaired adipogenesis in hypertrophic obesity in man. Am J Physiol Endocrinol Metab. 2009; 297:999–1003. 10.1152/ajpendo.00377.2009 19622783

[3] Isakson P, Hammarstedt A, Gustafson B, Smith U. Impaired preadipocyte differentiation in human abdominal obesity: role of Wnt, tumor necrosis factor-alpha, and inflammation. Diabetes. 2009; 58:1550–1557. 10.2337/db08-1770 19351711 PMC2699851

[4] Vidal-Puig A. Adipose tissue expandability, lipotoxicity and the metabolic syndrome. Endocrinol Nutr. 2013; 60:39–43. 24490226 10.1016/s1575-0922(13)70026-3

[5] Cinti S, Mitchell G, Barbatelli G, Murano I, Ceresi E, Faloia E, et al Adipocyte death defines macrophage localization and function in adipose tissue of obese mice and humans. J Lipid Res. 2005; 46:2347–55. 10.1194/jlr.M500294-JLR200 16150820

[6] Weyer C, Foley JE, Bogardus C, Tataranni PA, Pratley RE. Enlarged subcutaneous abdominal adipocyte size, but not obesity itself, predicts type II diabetes independent of insulin resistance. Diabetologia. 2000; 43:1498–506. 10.1007/s001250051560 11151758

[7] McLaughlin TM, Liu T, Yee G, Reaven GM, Tsao P, Cushman SW, Sherman A. et al Pioglitazone increases the proportion of small cells in human abdominal subcutaneous adipose tissue. Obesity 2010; 18:926–31. 10.1038/oby.2009.380 19910937 PMC9413023

[8] Wang Y, Rimm EB, Stampfer MJ, Willett WC, Hu FB. Comparison of abdominal adiposity and overall obesity in predicting risk of type 2 diabetes among men. Am. J. Clin. Nutr. 2005; 81: 555–563. 15755822 10.1093/ajcn/81.3.555

[9] Carey VJ, Walters EE, Colditz GA, Solomon CG, Willett WC, Rosner BA, Body fat distribution and risk of non-insulin-dependent diabetes mellitus in women. The Nurses' Health Study. Am. J. Epidemiol. 1997; 145: 614–619. 9098178 10.1093/oxfordjournals.aje.a009158

[10] Tran TT, Yamamoto Y, Gesta S, Kahn CR. Beneficial Effects of Subcutaneous Fat Transplantation on Metabolism. Cell Metabolism. 2008; 7: 410–420. 10.1016/j.cmet.2008.04.004 18460332 PMC3204870

[11] Abate N, Garg A, Peshock RM, Stray-Gundersen J, Grundy SM. Relationships of generalized and regional adiposity to insulin sensitivity in men. J Clin Invest. 1995; 96: 88–98. 10.1172/JCI118083 7615840 PMC185176

[12] Liu A, McLaughlin T, Liu T, Morton J, Cushman SW, Reaven GM, Tsao PS. et al Differential intra-abdominal adipose tissue profiling in obese, insulin-resistant women. Obes Surg. 2009; 19:1564–73. 10.1007/s11695-009-9949-9 19711137 PMC3181138

[13] Klöting N, Fasshauer M, Dietrich A, Kovacs P, Schön MR, Blüher M. et al Insulin-sensitive obesity. Am J Physiol Endocrinol Metab. 2010; 299:E506–15. 10.1152/ajpendo.00586.2009 20570822

[14] Lumeng CN, Deyoung SM, Saltiel AR. Macrophages block insulin action in adipocytes by altering expression of signaling and glucose transport proteins. Am J Physiol Endocrinol Metab. 2007; 292: E166–E174. 10.1152/ajpendo.00284.2006 16926380 PMC3888778

[15] Uysal KT, Wiesbrock SM, Marino MW, Hotamisligil GS. Protection from obesity-induced insulin resistance in mice lacking TNF-alpha function. Nature. 1997; 389: 610–4. 10.1038/39335 9335502

[16] Rodbell M. Metabolism of isolated fat cells. Effects of hormones on glucose metabolism and lipolysis. J Biol Chem. 1964; 239:375–380. 14169133

[17] Mehta R, Birerdinc A, Hossain N, Afendy A, Chandhoke V, Baranova A.et al Validation of endogenous reference genes for qRT-PCR analysis of human visceral adipose samples. BMC Mol Biol. 2010; 11–39. 20492695 10.1186/1471-2199-11-39 PMC2886049

[18] Livak KJ, Schmittgen TD. Analysis of relative gene expression data using real-time quantitative PCR and the 2(-Delta Delta C(T)) Method. Methods. 2001; 25: 402–408. 10.1006/meth.2001.1262 11846609

[19] Weisberg SP, McCann D, Desai M, Rosenbaum M, Leibel RL, Ferrante AW Jr. et al Obesity is associated with macrophage accumulation in adipose tissue. J Clin Invest. 2003; 112:1796–1808. 10.1172/JCI19246 14679176 PMC296995

[20] Lumeng CN, Bodzin JL, Saltiel AR. Obesity induces a phenotypic switch in adipose tissue macrophage polarization. J Clin Invest. 2007; 117:115–184. 10.1172/JCI29881 PMC1716210 17200717

[21] Liu LS, Spelleken M, Rohrig K, Hauner H, Eckel J. Tumor necrosis factor-alpha acutely inhibits insulin signaling in human adipocytes:implication of the P80 tumor necrosis factor receptor. Diabetes 1998; 47:515–522. 9568681 10.2337/diabetes.47.4.515

[22] Hotamisligil GS, Murray DL, Choy LN, Spiegelman BM. Tumor necrosis factor alpha inhibits signaling from the insulin receptor. Proc Natl Acad Sci USA. 1994; 91:4854–4858. 8197147 10.1073/pnas.91.11.4854 PMC43887

[23] Lacasa D, Taleb S, Keophiphath M, Miranville A, Clement K. Macrophage-secreted factors impair human adipogenesis: involvement of proinflammatory state in preadipocytes. Endocrinology. 2007; 148:868–77. 10.1210/en.2006-0687 17082259

[24] Couturier J, Patel SG, Iyer D, Balasubramanyam A, Lewis DE. Human monocytes accelerate proliferation and blunt differentiation of preadipocytes in association with suppression of C/EBPΑ mRNA. Obesity. 2012; 20:253–62. 10.1038/oby.2011.275 21869759 PMC4364279

[25] Chazenbalk G, Bertolotto C, Heneidi S, Jumabay M, Trivax B, Aronowitz J et al Novel pathway of adipogenesis through cross- talk between adipose tissue macrophages, adipose stem cells and adipocytes: evidence of cell plasticity. PLoS One. 2011; 6:e17834 10.1371/journal.pone.0017834 21483855 PMC3069035

[26] Macotela Y, Emanuelli B, Mori MA, Gesta S, Schulz TJ, Kahn CR. et al Intrinsic differences in adipocyte precursor cells from different white fat depots. Diabetes. 2012; 61:1691–9. 10.2337/db11-1753 22596050 PMC3379665

[27] Adams M, Montague CT, Prins JB, Lazar MA, Chatterjee VK, O'Rahilly S. et al Activators of peroxisome proliferator-activated receptor gamma have depot-specific effects on human preadipocyte differentiation. J Clin Invest. 1997;1003:149–53. 10.1172/JCI119870 PMC508528 9399962

[28] Sewter CP, Blows F, Vidal-Puig A, O'Rahilly S. Regional differences in the response of human pre-adipocytes to PPARgamma and RXRalpha agonists. Diabetes. 2002; 51:718–23. 11872672 10.2337/diabetes.51.3.718

[29] Perrini S, Laviola L, Cignarelli A, Melchiorre M, De Stefano F, Caccioppoli C, Fat depot-related differences in gene expression, adiponectin secretion, and insulin action and signalling in human adipocytes differentiated in vitro from precursor stromal cells. Diabetologia. 2008; J51:155–64. 10.1007/s00125-007-0841-7 17960360

[30] Russo V, Yu C, Belliveau P, Hamilton A, Flynn LE. Comparison of human adipose-derived stem cells isolated from subcutaneous, omental, and intrathoracic adipose tissue depots for regenerative applications. Stem Cells Transl Med. 2014;3:206–17. 10.5966/sctm.2013-0125 24361924 PMC3925056

[31] Perrini S, Ficarella R, Picardi E, Cignarelli A, Barbaro M, Nigro P et al Differences in gene expression and cytokine release profiles highlight the heterogeneity of distinct subsets of adipose tissue-derived stem cells in the subcutaneous and visceral adipose tissue in humans. PLoS One. 2013; 8: e57892 10.1371/journal.pone.0057892 23526958 PMC3589487

[32] Baglioni S, Cantini G, Poli G, Francalanci M, Squecco R, Di Franco A et al Functional differences in visceral and subcutaneous fat pads originate from differences in the adipose stem cell. PLoS One. 2012; 7: e36569 10.1371/journal.pone.0036569 22574183 PMC3344924

[33] Suganami T, Nishida J, Ogawa Y. A paracrine loop between adipocytes and macrophages aggravates inflammatory changes. Arterioscler Thromb Vasc Biol. 2005; 25:2062–2068. 10.1161/01.ATV.0000183883.72263.13 16123319

