# Supplementary figures and images for: Adipose tissue macrophages impair preadipocyte differentiation in humans
Source: PLoS One. 2017 Feb 2;12(2):e0170728. doi: 10.1371/journal.pone.0170728 (PMC5289462; doi:10.1371/journal.pone.0170728)

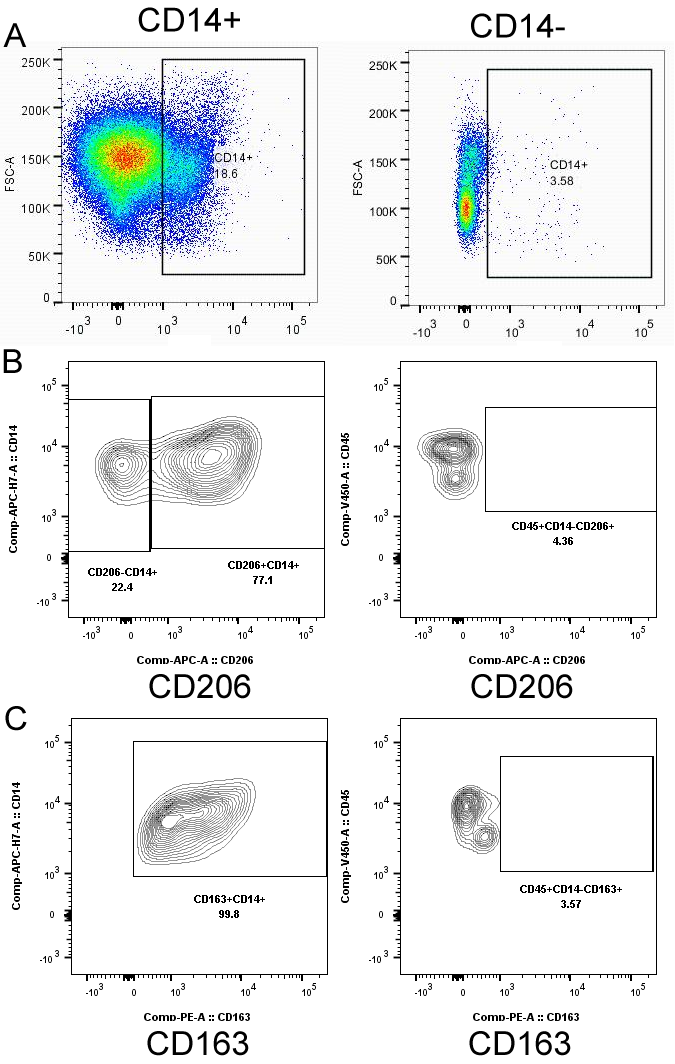

Supplement: S1 Fig — (a) Representative FACS analysis perform on SVCs in the presence or absence of CD14 positive cells from human adipose tissue using CD14(APC-H7) and CD45 (V450) antibodies. (b-c) Characterization of the isolated CD14+ cells. To characterize the CD14+ cells population, FACS analysis was performed on the freshly isolated CD14 positive cells from SVC. (b, c) All cells express both CD206 and CD163macrophage specific markers. (TIF) [file pone.0170728.s001.tif]

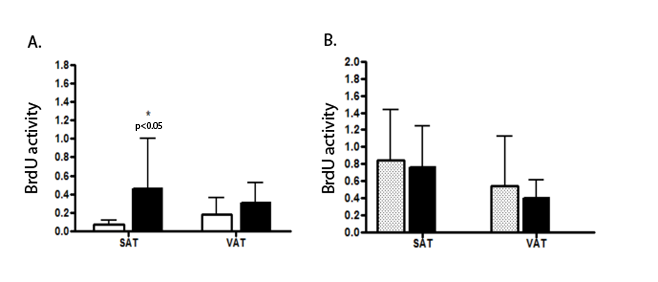

Supplement: S2 Fig — (A) Rate of proliferation of preadipocytes after 2 days of coculture with Cd14+ cells. Rate of proliferation was determined by BrdU incorpartion N = 3, error bars are means ± s.d., *P < 0.05 compared to CD14- cells. (B) Rate of proliferation of preadipocytes during coculture with CD14 + cells at day 7 at a 1:100, CD14+ cells: preadipocyte ratio. N = 3, error bars are means ± s.d. (TIF) [file pone.0170728.s002.tif]

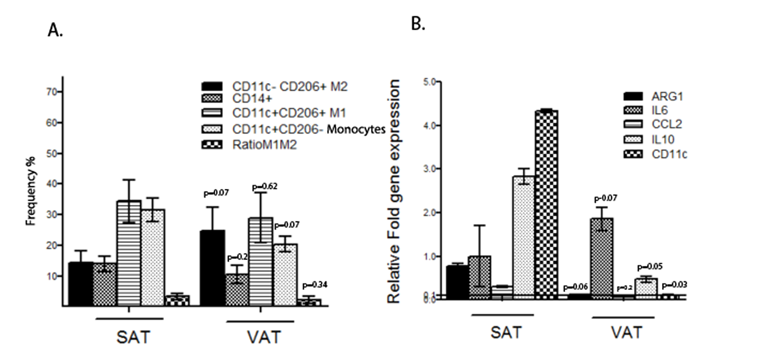

Supplement: S3 Fig — (A) Characterization of M2 and M1 phenotype by flow cytometry in SAT and VAT (N = 6, data are presented as mean ± SD). (B) Relative fold change in gene expression of M2 (ARG1, IL10) and M1 (CD11c, IL6 and CCL2) markers in SAT and VAT (N = 7, data are presented as mean ± SD) (TIF) [file pone.0170728.s003.tif]
